# Characterization of bacterial community dynamics dominated by salinity in lakes of the Inner Mongolian Plateau, China

**DOI:** 10.3389/fmicb.2024.1448919

**Published:** 2024-08-21

**Authors:** Guo Xin, Shi Xiaohong, Shi Yujiao, Li Wenbao, Wang Yanjun, Cui Zhimou, Lauri Arvolab

**Affiliations:** ^1^Water Conservancy and Civil Engineering College, Inner Mongolia Agricultural University, Hohhot, China; ^2^Inner Mongolia Key Laboratory of Protection and Utilization of Water Resources, Hohhot, China; ^3^State Gauge and Research Station of Wetland Ecosystem, Wuliangsuhai Lake, Bayan Nur, China; ^4^Lammi Biological Station, Ecosystems and Environment Research Programme, Faculty of Biological and Environmental Sciences, Helsinki University, Helsinki, Finland

**Keywords:** bacteria, community composition, community assembly processes, co-occurrence network, Badanjilin Desert

## Abstract

Microorganisms in lakes are sensitive to salinity fluctuations. Despite extensive prior research on bacterial communities, our understanding of their characteristics and assembly mechanisms in lakes, especially in desert lakes with different salinities. To address this issue, we collected three samples from freshwater lakes, six from brackish lakes, and five from salt lakes in the Badanjilin Desert. The 16S rRNA gene sequencing was applied to investigate the bacterial interactions with rising salinity, community coexistence patterns, and assembly mechanisms. Our findings suggested that the increased lake salinity significantly reduces the bacterial community diversity and enhanced the community differentiation. Significant variations were observed in the contribution of biomarkers from Cyanobacteria, Chloroflexi, and Halobacterota to the composition of the lake bacterial communities. The bacterial communities in the salt lakes exhibited a higher susceptibility to salinity limitations than those in the freshwater and brackish lakes. In addition, the null modeling analyses confirmed the quantitative biases in the stochastic assembly processes of bacterial communities across freshwater, brackish, and saline lakes. With the increasing lake salinity, the significance of undominated and diffusion limitation decreased slightly, and the influence of homogenizing dispersal on community assembly increased. However, the stochasticity remained the dominant process across all lakes in the Badanjilin Desert. The analysis of co-occurring networks revealed that the rising salinity reduced the complexity of bacterial network structures and altered the interspecific interactions, resulting in the increased interspecies collaboration with increasing salinity levels. Under the influence of salinity stress, the key taxon Cyanobacteria in freshwater lakes (*Schizothrix_LEGE_07164*) was replaced by Proteobacteria (*Thalassobaculum* and *Polycyclovorans*) in brackish lakes, and Thermotogota (*SC103*) in salt lakes. The results indicated the symbiotic patterns of bacterial communities across varying salinity gradients in lakes and offer insights into potential mechanisms of community aggregation, thereby enhancing our understanding of bacterial distribution in response to salinity changes.

## 1 Introduction

Microorganisms, encompassing diverse types and ubiquitous distribution, play a crucial role in nearly all of Earth’s physicochemical cycles. Within lake ecosystems, bacteria are integral components of the microbial food web and serve as sensitive indicators of changes in the trophic status, reflecting alterations in lake health and environmental conditions (Newton et al., 2011; Pernthaler, 2017). In recent years, owing to the significant role of bacteria in lake ecosystems, there has been a growing emphasis on microbiome studies concerning the ecological structure and evolutionary processes of freshwater bacteria. The comprehensive investigations of the bacterial communities in lakes have substantially enhanced our understanding of microbial communities (Jakob, 2005; Williamson et al., 2009; Xiao et al., 2020). These investigations have demonstrated that the differences in habitat characteristics can significantly affect the bacterial community succession (Dang et al., 2022). For example, Gao et al. observed higher α-diversity in sediment bacterial communities than in water column bacterial communities (Gao et al., 2023). Zhang et al. identified the dual regulation of the bacterial community on the Qinghai-Tibet Plateau by salinity and season (Zhang J. et al., 2022; Zhang X. L. et al., 2022). However, most recent studies have focused on individual lakes without systematically comparing the dynamics of bacterial communities across diverse environmental contexts. This has limited our understanding of variability in the structure and assembly processes of bacterial communities.

In recent years, with a warming climate and increased evaporation from lakes, salt enrichment that is compounded by anthropogenic activities has accelerated the changes in the environmental conditions of aquatic ecosystems (Paerl et al., 2018). The area of inland lakes with high salt contents has increased, now encompassing approximately 44% of the total inland lake area (Messager et al., 2016; Zhang J. et al., 2022; Zhang X. L. et al., 2022). Salinity generally exerts a confining effect on aqueous substances and creates substantial environmental disparities across lakes with varying salinity levels. Consequently, bacterial communities exhibit rapid responses to nutrient changes, altering the ecosystem by reshaping the composition and assembly patterns of the biological regions (Zhang J. et al., 2021; Zhang X. et al., 2021). Numerous studies have demonstrated that both stochastic and deterministic mechanisms contribute to the variations in the biogeographical patterns of microorganisms (David et al., 2021; Jing et al., 2023). Deterministic processes indicate the significance of biotic (microbial interactions) and abiotic (nutrient availability) factors (Pholchan et al., 2013), which hypothesizes that microbial β-diversity is primarily influenced by the composition of the microbial community by environmental selection, including both homogeneous and heterogeneous selection. In contrast, stochastic processes demonstrate that species dynamics are not determined by competitive disparities but are governed by stochastic elements such as dispersal limitation, homogeneous dispersal, and undominated factors in community dynamics (Zhou and Ning, 2017). Researchers are striving to explore whether ecological processes mediated by bacteria conform to specific environmental gradients. However, they acknowledged that the differences in habitat types and microbial taxa may be in constant flux (Tang et al., 2020), rendering the mechanisms of community construction ambiguous. For example, the bacterial community formation in the Taihu Lake estuary is primarily dominated by stochastic processes, notably dispersal limitations (Jiang et al., 2024). Furthermore, the deterministic contribution of ecological assembly of soil bacterial communities has decreased in different watersheds (Jiao et al., 2020; Tang et al., 2020; Fodelianakis et al., 2021). Therefore, further exploration is required to quantify the contributions of various habitat conditions such as rising salinity to the deterministic and stochastic processes and ecological networks, to find a unified framework for describing the relative significance of the controlling factors, and in this way to improve the comprehensive prediction of the impacts of microbial ecological processes in lakes, and to provide an effective biodiversity conservation policies.

The sensitivity of bacterial communities to their environment alters their bioconcentration processes (Abrantes et al., 2006; Hahn, 2006). Additionally, the ecological niche theory proposes that the relative ratios of different ecological assembly processes vary depending on the strength of environmental filtration (Goddard and Whittle, 2015). Various ecological selections, dispersal, and drifting effects can further lead to microbial communities exhibiting diverse metabolic profiles. We revealed the interactions and complexity of environmental bacteria by leveraging co-occurrence network relationships (Hu et al., 2017; Hui et al., 2022). Moreover, salinity that can be pivotal in governing the microbial dynamics in lake ecosystems is acknowledged as a significant factor that strongly influences microbial states. Hence, the environmental fluctuations associated with salinity may directly affect the functional stability mediated by the bacteria in lake ecosystems (Yannarell and Paerl, 2007; Egamberdieva et al., 2010). It is crucial to understand how the ecological relationships and interspecific interactions of microorganisms in lakes with varying salinities respond to changes in salinity. Although habitat conditions can significantly affect the dynamic mechanisms of the bacterial community assembly in lakes, numerous studies have only examined the impact of salt levels on the bacterial community composition in individual lakes. Few investigations have explored the symbiotic patterns and assembly mechanisms of bacterial communities across large spanning salinity gradients, causing a lack of systematic comparisons of study results. The Alashan region in northern China is characterized by scarce precipitation and significant climatic differences, which leads to large variations in salinity in different lakes, making it an ideal location for our study.

In this study, we examined the microbial community characteristics, focusing particularly on salinity distribution within lakes, with the aim of addressing the existing knowledge gaps. This report outlines the bacterial diversity observed in 14 lakes located in the Badanjilin Desert, Inner Mongolia, China, covering salinity levels from 0.88–168.15. The 14 lakes were categorized as freshwater (A7, A8, and A14), brackish (A1, A2, A6, A9, A10, and A13), or saltwater (A3, A4, A5, A11, and A12). Using the 16S rRNA gene high-throughput sequencing, we investigated the bacterial community structure, assembly processes, and molecular ecological network structures in lake ecosystems across varying salinity gradients in the Inner Mongolia Plateau. The objectives of this study were (1) to uncover and compare the alterations in the bacterial community diversity and composition across salinity gradients, (2) to examine the variations in the bacterial community assembly processes along these gradients, and (3) to assess interspecies interactions across salinity gradients, with the aim of understanding differences in bacterial community responses within freshwater, brackish water, and salt lake ecosystems.

## 2 Materials and methods

### 2.1 Study lake and sample collection

**Figure 1:**
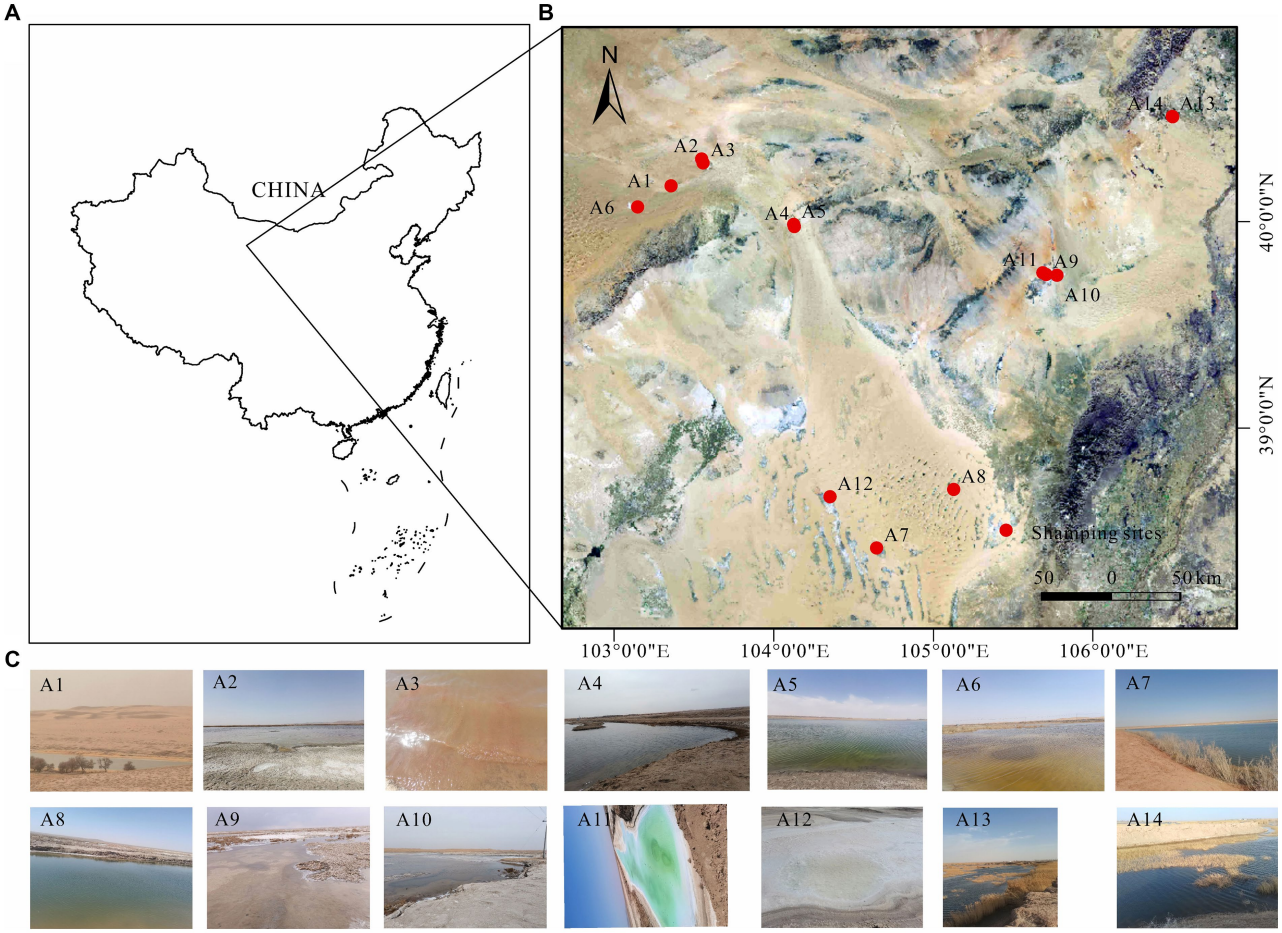
**(A)** Geographic location of Badanjilin Desert in Alashan, Inner Mongolia, China, **(B)** map of lake sampling sites in Badanjilin Desert, and **(C)** live photos of each lake.

The Badanjilin Desert, situated in the northwestern part of the Alashan Plateau in China, lies north of the Yabulai and Heishantou Mountains (Zhang et al., 2023). Bordered by mountain ranges to the south and southeast and encompassing low-lying areas of the Gulinai grasslands and the Guezhi Lake plains, it spans an area of approximately 50,000 km^2^, ranking as the fourth largest desert in China (Dong et al., 2004). Characterized by an extreme continental climate, the region experiences scorching summers and cold, dry winters, with annual rainfall ranging from 68 mm to 172 mm (Gates et al., 2008; Zhang J. et al., 2021; Zhang X. et al., 2021). The samples were collected in July 2023 from three freshwater lakes (A7, A8, and A14, salinity ≤1 g/L), six brackish lakes (A1, A2, A6, A9, A10, and A13, 1 g/L < salinity ≤50 g/L), and five salt lakes (A3, A4, A5, A11, and A12, salinity >50 g/L) (Figure 1). A preliminary survey was conducted before the main study began. All the freshwater, brackish, and salt lakes are situated in the Badanjilin Desert, and precipitation is the primary source of recharge for these lakes (Yang et al., 2010; Zhang et al., 2023). They are all minimally affected by human activities, with salinity variations presenting the greatest distinction. This offers an ideal opportunity to investigate the effects of varying salinity gradients on bacterial community characterization. At each sampling site, 2 L of the water samples were collected using specialized devices, and prewashed sterile polyethylene bottles were adopted for the collection. Various physicochemical indicators were measured during sample collection. Collected samples were stored in tanks for refrigeration and quickly transported to the laboratory for processing. All water samples were filtered through a 0.22 μm polycarbonate membrane and stored at −80° C for subsequent DNA extraction. Concurrently, samples were stored at 4°C for the determination of their physicochemical properties, and subsequent laboratory processing was completed within 12 h of the end of each sample collection.

### 2.2 Environmental information

The salinity of the samples was determined using a mobile multiparameter water quality monitor. In addition, the pH, salinity (SAL), and dissolved oxygen (DO) were measured using the same device. Nutrient concentrations, including free-state ammonia nitrogen (NH_4_^+^-N), chlorophyll-a (Chl a), total nitrogen (TN), total phosphorus (TP), dissolved inorganic phosphorus (DIP), total organic matter (COD), and total dissolved phosphorus (DTP), were analyzed in the laboratory using standard methods (GB 3838-2002) (Shi et al., 2023) (Supplementary Figure S1).

### 2.3 DNA extraction and processing of sequencing data

Microbial community genomic DNA was extracted from water samples following the manufacturer’s instructions. The DNA extract was checked on 1% agarose gel, and DNA concentration and purity were determined with NanoDrop 2000 UV–vis spectrophotometer (Thermo Scientific, Wilmington, United States). The hypervariable region of the bacterial 16S rRNA gene were amplified with primer pairs 515F (5’-GTGCCAGCMGCCGCGGGTAA-3′) and 806R (5’-GGACTACVSGGTATCTAAT-3′) by an ABI GeneAmp^®^ 9,700 PCR thermocycler (ABI, CA, United States) (Shi et al., 2023). The PCR products extracted from 2% agarose gels were purified using a PCR clean-up kit and quantified using a Qubit 4.0 (Thermo Fisher Scientific, United States) according to the manufacturer’s protocol. Purified amplicons were pooled in equimolar and paired-end sequenced on an Illumina MiSeq PE300 platform/NovaSeq PE250 platform (Illumina, San Diego, United States) according to the standard protocols by Majorbio Bio-Pharm Technology Co., Ltd. (Shanghai, China). The obtained sequences were quality-checked by filtering with fastp (0.19.6) and merged with FLASH (v1.2.11) (Mago and Salzberg, 2011; Chen et al., 2018). The sequences were denoised for the high quality using the DADA2 plugin within the Qiime2 (version 2020.2) pipeline, utilizing the recommended parameters to achieve the single-nucleotide resolution based on the error curves within the samples (Callahan et al., 2016; Bolyen et al., 2019). The DADA2 denoised sequences are commonly known as Amplicon Sequence Variants (ASV).

### 2.4 Data analysis

Venn diagrams were created to tally and visualize the number of common and unique ASVs across different environmental samples using R language tools (version 3.3.1). α-Diversity indices, such as the Chao index, Shannoneven index, and Shannon index, were computed using the Mothur software (version v.1.30.2), with ASVs clustered at a 97% similarity level for index evaluation. The Kruskal-Wallis rank sum test was used for statistical analyses (Wang et al., 2022). The correlations among the microbial community similarities, diversity, and salinity were assessed using the vegan package (2.4.3) in R (version 3.3.1) (Yi et al., 2021). The distinctiveness of bacterial community composition in the freshwater, brackish, and salt lakes was assessed using Non-Metric Multidimensional Scaling (NMDS) with ASV-based Bray-Curtis Distance (Ziegler et al., 2017), and the R language (version 3.3.1) was utilized for drawing. Moreover, the LEfSe Analysis was employed to conduct the all-to-all (more stringent) difference tests at both the phylum and genus levels to analyze differential species among freshwater, brackish water, and saline lakes. On the basis of statistical significance (*p* < 0.05), phyla and genera with LDA values greater than 4 were selected as potential biomarkers, the magnitude of the species influence on the observed differences was measured using the LDA value, indicating a potentially crucial role of these species in environmental change processes (Liu et al., 2022). The impacts of environmental factors on the bacterial community composition in the investigated water bodies was studied using the R language vegan package (version 2.4.3), employing the analysis of the RDA and pheatmap. The goodness-of-fit statistic (*R*^2^) (*p* < 0.05) was adopted to evaluate the relative importance of the different environmental factors in explaining community variation.

### 2.5 Network analysis

To explore the molecular ecological network structure of bacterial communities across varying salinity gradients, Networkx (version 1.11) was utilized to calculate all potential correlations between genera. Statistically significant genera, determined using the Spearman method, were integrated into the network analysis. Using Gephi (version 0.9.2), the networks were constructed for the genera in the freshwater, brackish water, and saline lakes. The visualization encompasses the assessment of the mean node degree, modularity, mean path length, network diameter, and network density, with edges randomly assigned to any node with equal probability (Lieberman et al., 2005). Subsequently, Zi (intra-module connectivity) and Pi (inter-module connectivity) were calculated based on the node characteristics, classifying nodes into Module hubs (Zi > 2.5 and Pi <0.62), Connectors (Zi < 2.5 and Pi >0.62), and Peripherals (Zi < 2.5 and Pi <0.62). The applications of Zi and Pi in the networks of coexisting microorganisms within lakes served as a foundation for identifying core microbial species in different habitats (Olesen et al., 2007).

### 2.6 Microbial community assembly processes

To advance our understanding of the microbial community assembly in the freshwater, brackish water, and saline lakes, we quantitatively inferred the community construction mechanisms through the phylogenetic split-box null model analysis implemented in the “Picante” package iCAMP in R (Iqbal et al., 2024). The observed ecological patterns were compared with randomly distributed patterns using null models, and the beta nearest taxon index (βNTI) was employed to characterize the turnover in the phylogenetic composition of the community. The normalization effect between the observations and the mean of the null model distribution was quantified using βNTI. The βNTI values represent the rates of phylogenetic turnover, where the values exceeding 2 denote the significantly higher turnover than expected, whereas the values below-2 denote the significantly lower turnover than expected, attributed to the heterogeneous and homogeneous selection by deterministic processes, respectively. Furthermore, the |βNTI| values less than 2 indicate the dominance of stochastic processes encompassing dispersal limitation, homogeneous dispersal, and undominated factors (Stegen et al., 2012; Dini-andreote et al., 2015).

## 3 Results

### 3.1 Patterns of bacterial distribution in lakes of different habitat types

**Figure 2:**
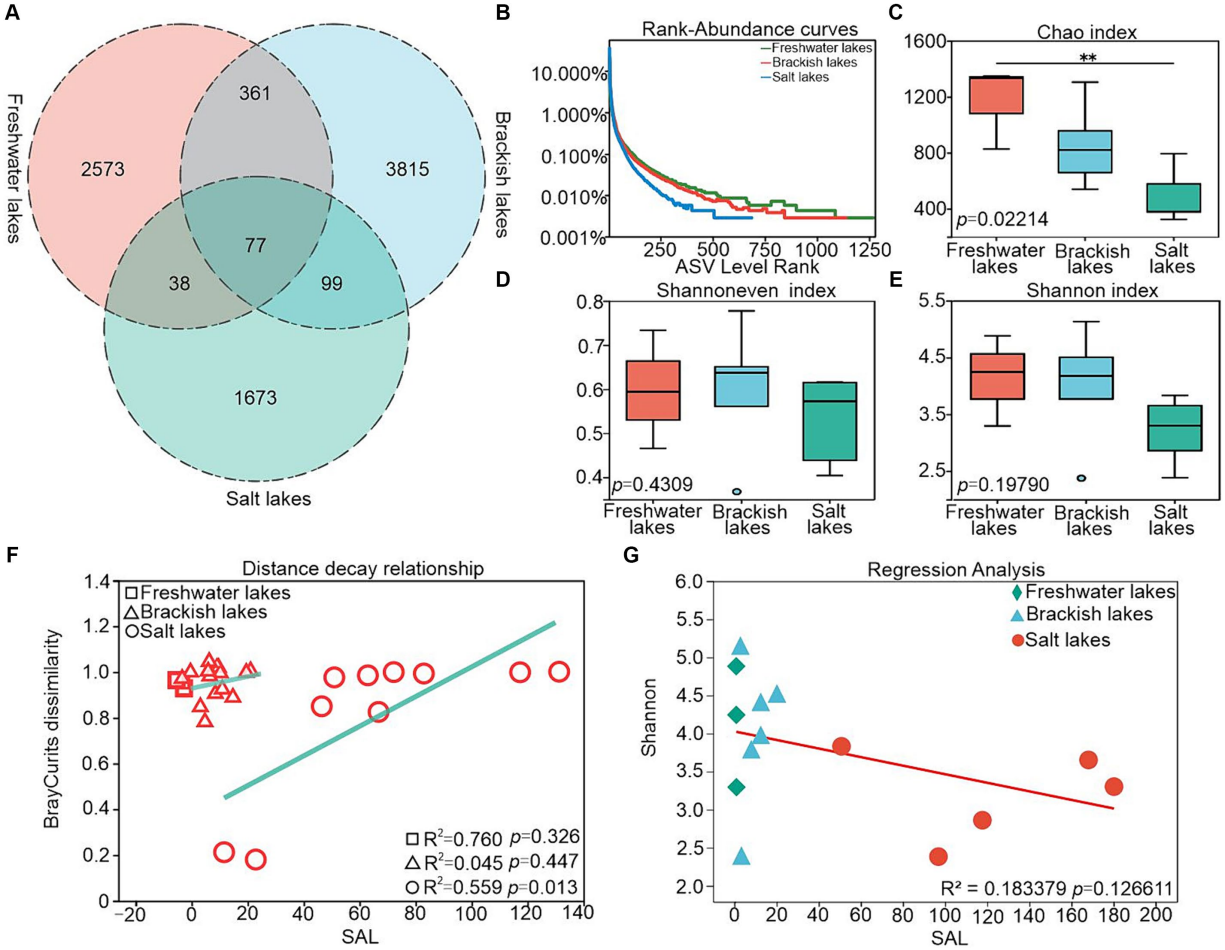
**(A)** Venn diagram showing shared and unique ASVs in freshwater, brackish and saline lakes, **(B)** rank-abundance curves, **(C)** Chao index responding to species abundance, **(D)** Shannoneven index responding to species uniformity, **(E)** Shannon index responding to species diversity, **(F)** relationship between SAL with Bray–Curtis dissimilarity, and **(G)** relationship between SAL with Shannon.

A total of 8,636 ASVs were identified across the freshwater, brackish water, and salt lake samples, with the ASV counts in individual samples ranging from 1887 to 4,352 (Figure 2A), where the salt lake exhibited the lowest count. Freshwater, brackish and salt lakes share only 77 ASVs, only 0.89% of the total number, suggesting that bacterial communities in lakes with different salinities vary considerably. The rank-abundance curves exhibited a gradual decline in species abundance across the freshwater, brackish, and saline lakes, which was consistent with the trend observed in the Chao index (Figures 2B,C). The Shannoneven index reached its peak in brackish water, indicating the most even distribution of species in this environment (Figure 2D). The species diversity remained relatively stable in freshwater and brackish lakes, both exhibiting Shannon values of approximately 4.00, whereas a significant decrease in species diversity was observed in saline lakes, with a Shannon value of approximately 3.20 (Figure 2E; Supplementary Table S1). This trend was further supported by the regression analysis between the salinity and species diversity (Figure 2G). Additionally, the Bray–Curtis dissimilarity of bacterial communities exhibited a positive correlation with the salinity differences, indicating that increasing salinity heightened community heterogeneity across salinity gradients (*R*^2^ = 0.559; *p* = 0.013) (Figure 2F).

### 3.2 Composition of microbial communities and discovery of biomarkers

**Figure 3:**
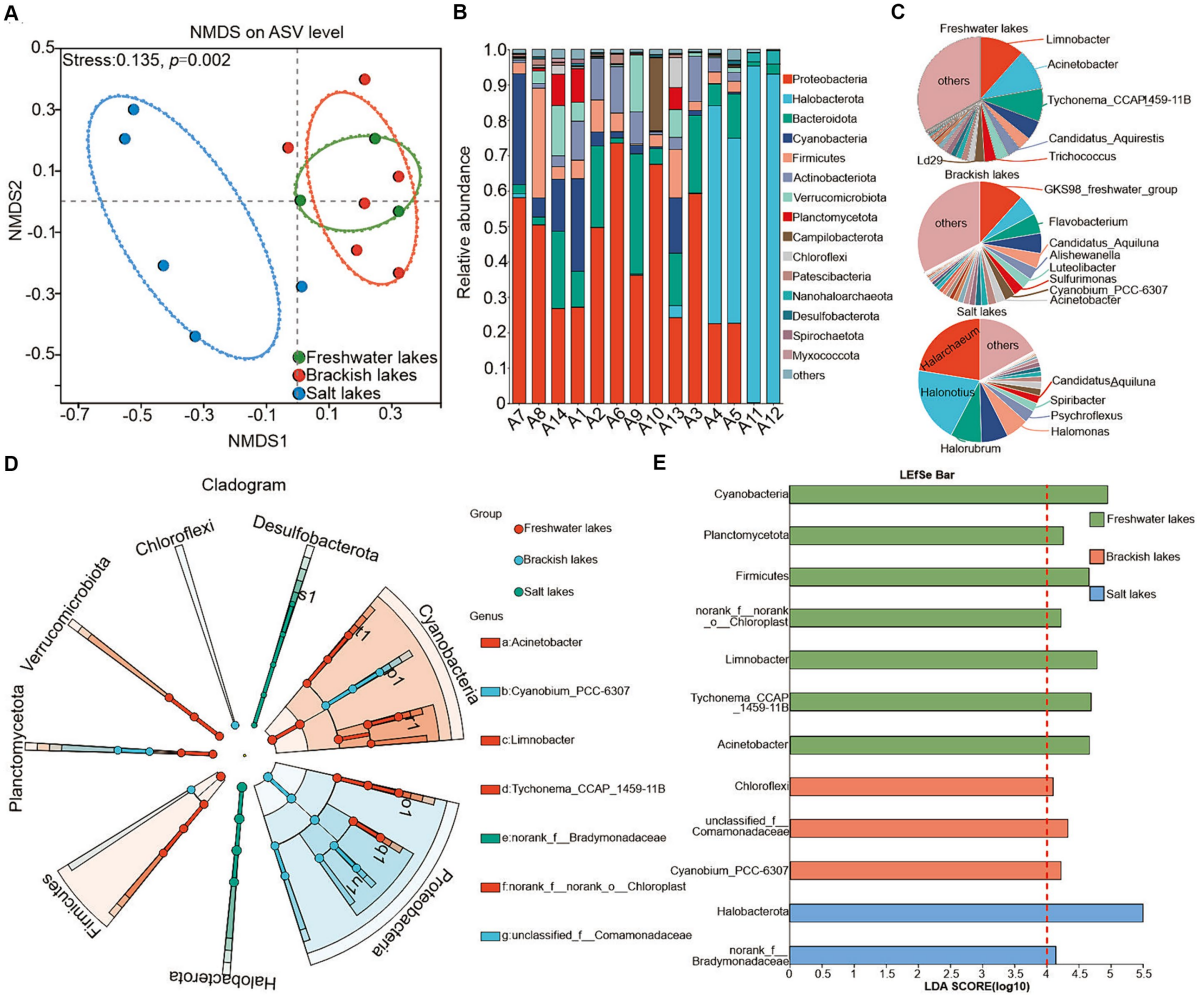
**(A)** Bray–Curtis similarity-based NMDS analysis of water column bacteria in different lake samples. Ellipses indicate 95% confidence intervals. Taxonomic composition of bacterial communities in lake samples at the phylum **(B)** and genus **(C)** levels, respectively, showing only dominant phyla and genera with mean relative abundance ≥1% and those already classified. In contrast, those with mean relative abundance <1% are classified as “other.” **(D,E)** To map the evolutionary branching of bacterial communities associated with freshwater, brackish, and saline lakes on the phylum and genus levels, the LDA value was set to 4. Higher scores indicate an increased influence of the relative abundance of species on the differential effect.

The principal coordinate analysis (PCoA) revealed significant differences between the bacterial communities in lakes with varying salinities (Figure 3A, *p* = 0.002). A total of 89 bacterial phyla were identified across all water samples. In the freshwater lakes, the dominant phyla included Proteobacteria (mean relative abundance of approximately 45.06%), Cyanobacteria (mean relative abundance of approximately 17.12%), and Firmicutes (mean relative abundance of approximately 12.59%). In the brackish water lakes, the dominant phyla were Proteobacteria (mean relative abundance of approximately 46.35%), Bacteroidota (mean relative abundance of approximately 14.62%), and Cyanobacteria (mean relative abundance of approximately 8.41%). In the Salt Lake, the dominant bacterial phyla shifted to Halobacterota (average relative abundance of approximately 60.45%), Proteobacteria (average relative abundance of approximately 20.90%), and Bacteroidota (average relative abundance of approximately 8.87%) (Figure 3B). Collectively, these dominant bacterial phyla accounted for approximately 70.00% of the total proportion across the lakes with various salinity gradients. Furthermore, the relative abundance of these dominant taxa varied significantly among lakes with different salinity gradients (Supplementary Table S2), confirming the distributional heterogeneity in the bacterial community composition of the water column, consistent with previous findings (Liu et al., 2022).

Figure 3C depicts the dominant microbial taxa at the genus level across the different sampling sites. In the freshwater lakes, *Limnobacter*, *Acinetobacter*, and *Tychonema_CCAP_1459-11B* were predominant, with average relative abundances of approximately 11.59, 10.59, and 8.58%, respectively. The community distribution of the brackish lakes highlighted *GKS98_freshwater_group*, *Flavobacterium*, and *Candidatus_Aquiluna*, with average relative abundances of approximately 11.70, 5.26, and 3.94%, respectively. In the salt lake, the dominant genera included *Halarchaeum* (22.41%), *Halonotius* (19.88%), and *Halorubrum* (8.13%), which were primarily influenced by salinity. The distribution patterns across the lakes presented various trends (Supplementary Table S3).

This study further explored high taxon biomarkers in the freshwater, brackish, and salt lakes using the least discriminant analysis (LDA) method. Significant variations in the dominant species of bacterial communities were observed across different salinity conditions. The LDA method identified eight phyla and seven genera (LDA > 4.0, *p* < 0.05) as potential biomarkers (Figure 3D). In the freshwater lakes, the enriched bacterial taxa included Cyanobacteria, Planctomycetota, Firmicutes, *Limnobacter*, *Tychonema_CCAP_1459-11B*, and *Acinetobacter*. Chloroflexi and *Cyanobium_PCC-6307* were prominent in the brackish lakes. In the salt lakes, Halobacterota exhibited the highest LDA scores, exerting the greatest influence on differences in species community composition. The identified species may play pivotal roles in environmental change processes (Figure 3E).

### 3.3 Factors driving bacterial community structure

**Figure 4:**
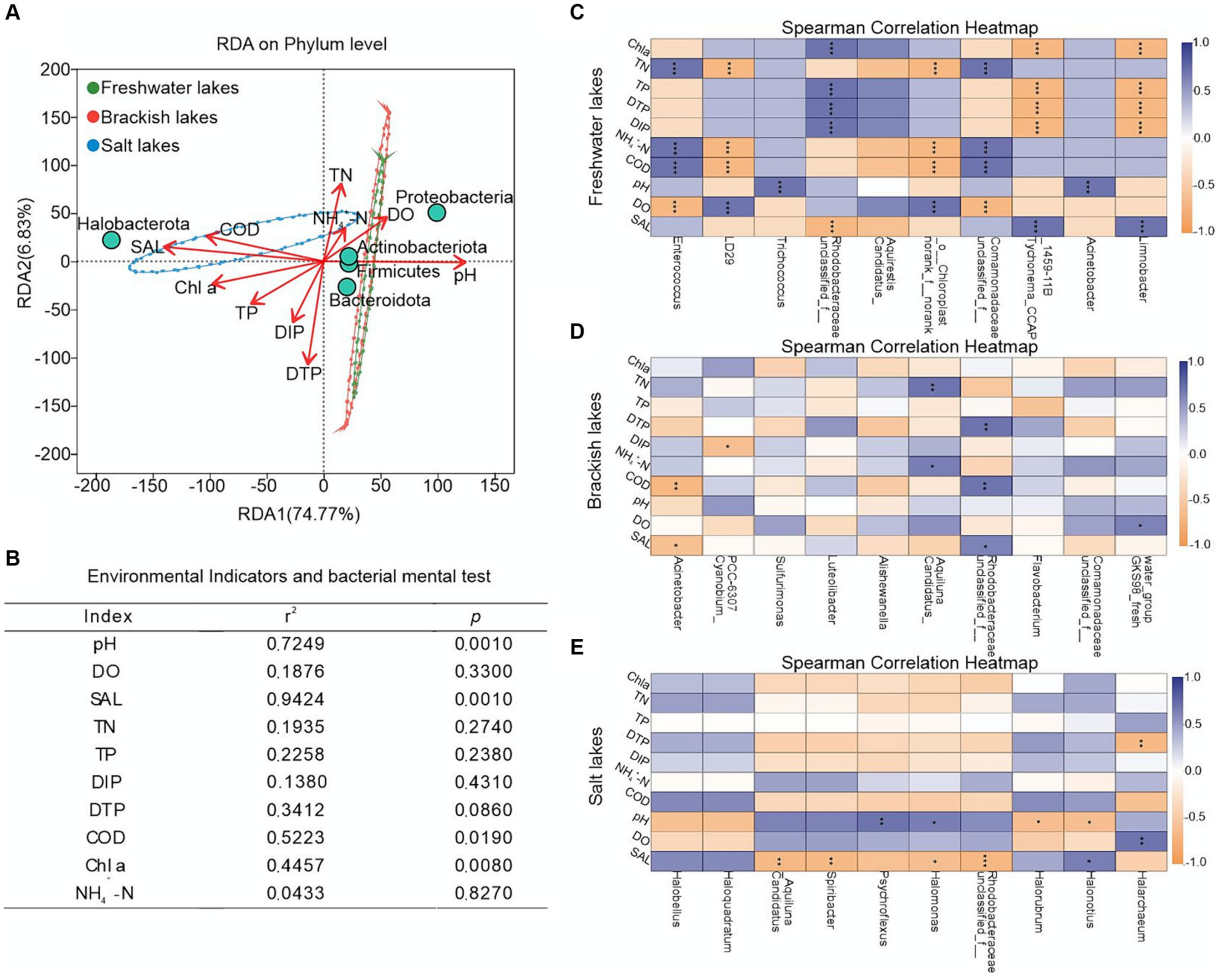
Impact of environmental indicators on microbial community composition in freshwater, brackish, and saline lake waters. **(A)** RDA ordination plot showing the significant environmental factors influencing the variation of bacterial community groups on the phylum level; **(B)** RDA analysis results, assessing the extent to explain the variation of bacterial community groups by environmental factors; **(C–E)** Spearman analysis was used to identify the major environmental factors controlling the variation in bacterial community groups at the genus level. The color gradient indicates the Spearman correlation coefficients, blue indicates the positive correlation, and yellow indicates the negative correlation coefficients. *, *p* < 0.05; **, *p* < 0.01; ***, *p* < 0.001.

To further understand the impact of environmental indicators on microbial community structure across varying salinity gradients, we conducted the RDA on the bacterial communities and environmental factors in the water bodies of the Badanjilin Desert. The results indicated that environmental factors accounted for 81.60% of the variation in the bacterial community structure (with 74.77% attributed to the RDA1 axis and 6.83% to the RDA2 axis) (Figure 4A). This suggested that the physicochemical parameters in the lakes with differing salinities effectively explained the variations in bacterial communities. Notably, pH, SAL, COD, and Chl a were significant factors influencing the changes in bacterial microbial community structure (*R*^2^ = 0.7249, *R*^2^ = 0.9424, *R*^2^ = 0.5223, and *R*^2^ = 0.4457, respectively; Figure 4B). pH significantly influenced the bacterial community structure in the freshwater and brackish lakes, promoting an increased relative abundance of Proteobacteria, Actinobacteriota, and Firmicutes. Conversely, the SAL, COD, and Chl a significantly (*p* < 0.05) affected the community structure of the saline lakes, positively correlating with the changes in the relative abundance of Halobacterota (Figure 4A).

Spearman correlation heatmap analysis revealed distinct environmental selection patterns among dominant genera across different habitats. In the freshwater lakes, pH exhibits significant positive correlations with *Trichococcus* (Firmicutes) and *Acinetobacter* (Proteobacteria). Conversely, Chl a was significantly negatively correlated with *Tychonema_CCAP_1459-11B* (Cyanobacteria) and *Limnobacter* (Proteobacteria), whereas COD led to inconsistent changes in the relative abundance of *Enterococcus* (Firmicutes) and *LD29* (Verrucomicrobiota) (Figure 4C). In the brackish lakes, COD notably suppressed the variation in the relative abundance of *Acinetobacter* (Figure 4D). The particularly noteworthy in saline lakes was the pronounced influence of environmental factors on Halobacterota, including *Halarchaeum*, *Halonotius*, and *Halorubrum* within this phylum (Figure 4E).

### 3.4 Mechanisms of bacterial community assembly in lakes with different salinity gradients

**Figure 5:**
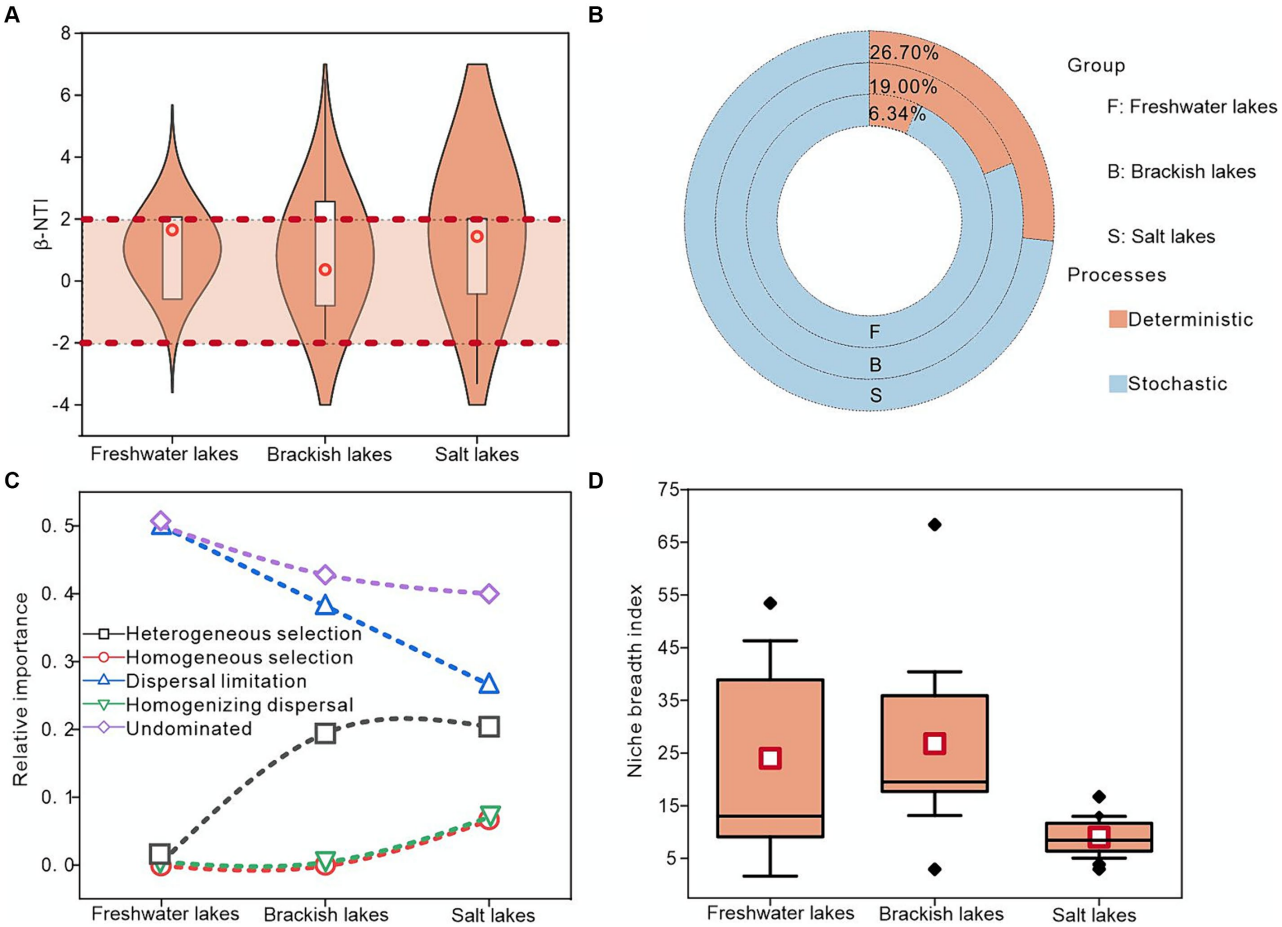
Exploring the mechanism of bacterial community assembly in freshwater, brackish, and saline lakes using null model analysis. **(A)** Distribution of β-NTI values in different samples, **(B)** relative contributions of deterministic and stochastic processes to bacteria in lakes with different salinities, **(C)** relative contributions of deterministic and stochastic ecological processes (stochastic processes including dispersal limitation, homogeneous dispersal, and undominated; and deterministic processes including heterogeneous selection and homogeneous selection), and **(D)** bacterial community ecological niche width.

First, the ecological niche widths of freshwater, brackish, and salt lakes were calculated, with the brackish lakes exhibiting the highest average widths of approximately 18.47. This suggested potentially lower species specialization and enhanced competitive ability within these lakes (Figure 5D). Second, the mechanism of bacterial community assembly across the freshwater, brackish, and salt lakes was investigated using a null model. These results indicated that community formation can be influenced by both deterministic and stochastic processes. Notably, approximately 93.66, 81.00, and 73.30% of the βNTI values in the freshwater, brackish water, and salt lakes, respectively, fell between-2 and 2, indicating the critical role of stochastic processes in the bacterial community assembly (Figures 5A,B). During the stochastic processes, the bacterial community assembly under various salinity gradients was primarily influenced by a larger proportion of undominated factors, particularly in the freshwater lakes where undominated factors contributed approximately 50.00%. Moreover, dispersal limitation played a more significant role in controlling bacterial community assembly across the freshwater, brackish water, and saline lakes than homogeneous dispersal. Notably, despite the dominance of stochastic processes, the importance of deterministic processes increased with salinity, with the heterogeneous selection processes exerting the highest contribution in the saline lakes at approximately 20.00% (Figure 5C). In summary, the stochastic processes predominantly governed the bacterial community assembly in lakes across different salinity gradients in the Badanjilin Desert, whereas the deterministic processes held more sway in salt lakes than in freshwater lakes.

### 3.5 Patterns of network distribution of bacterial contributions in different types of lakes

**Figure 6:**
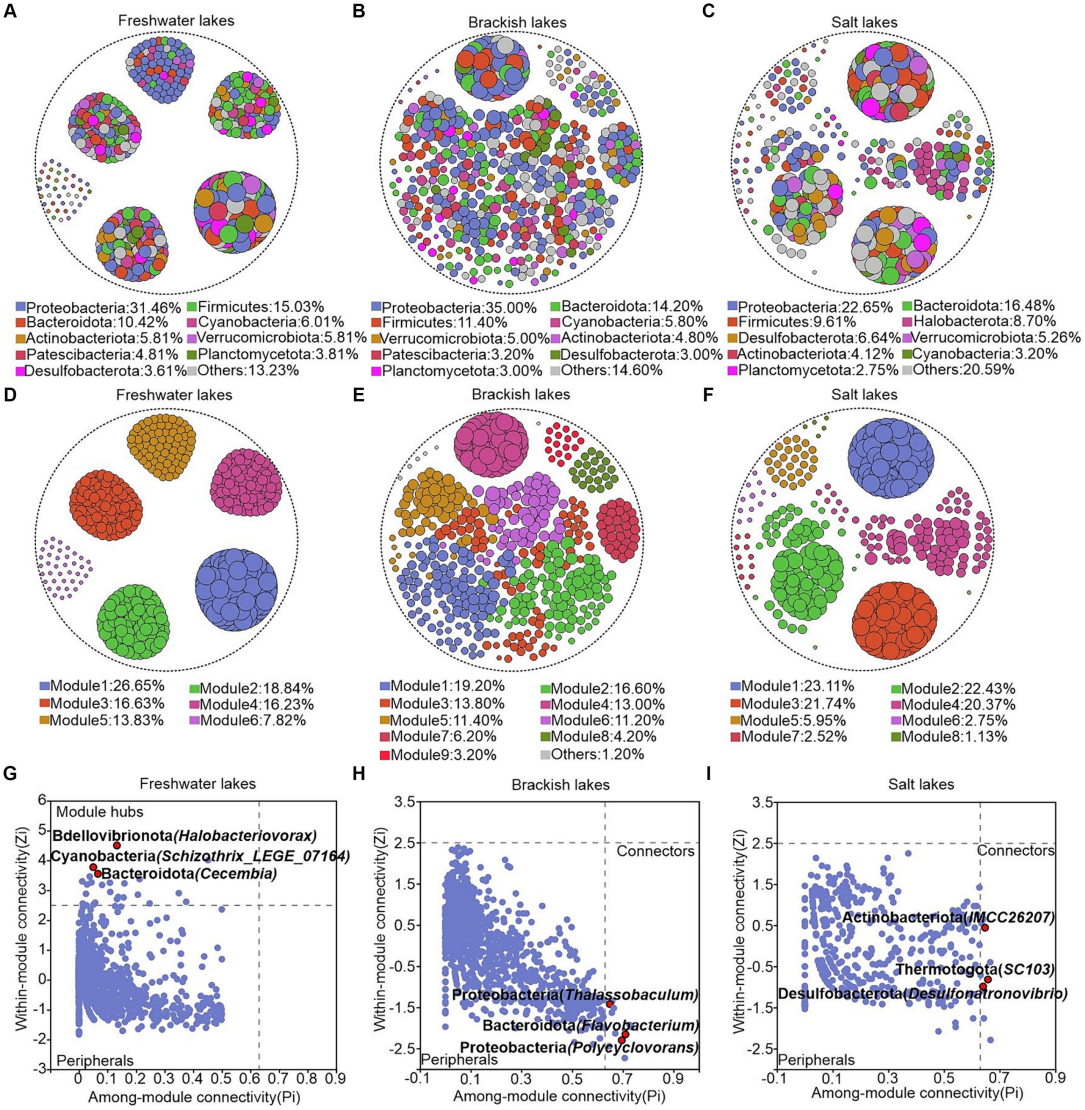
**(A–F)** Ecological network structure of freshwater, brackish, and saline lakes at the bacterial genus level. Node size is positively proportional to the degree value of the genus represented by that node, and nodes appearing in the graph are statistically significant (*p* < 0.05). **(A–C)** Network nodes are colored according to the bacterial phylum to which they belong, and **(D–F)** network nodes are colored according to the module to which they belong. **(G–I)** Module hubs (zi > 2.5, Pi ≤0.62) and connectors (zi ≤ 2.5, Pi >0.62). The name of each phylum is followed by parentheses representing the genera belonging to that phylum, and only the categorized genera are labeled in the figure.

We constructed the ecological network structures of bacterial communities in the freshwater, brackish, and saltwater lakes to investigate the effects of different environmental conditions on microbial interactions in the lake ecosystems. The results revealed that the freshwater lakes harbor a bacterial network structure comprising 22,880 edges, with a graph density of 0.184. The brackish lakes exhibited a structure with 7,984 edges and a graph density of 0.064, whereas salt lakes demonstrated a structure with 14,281 edges and a graph density of 0.15. Notably, the freshwater lakes exhibited a higher number of edges in their bacterial network structure than brackish and saline lakes, providing the direct evidence of more intricate patterns of network structure in freshwater ecosystems (Supplementary Table S4). In the lakes across varying salinity gradients, collaborative relationships predominantly shaped the interactions between bacterial genera, accounting for 64.55, 75.75, and 95.50% of positive correlations in the freshwater, brackish, and saline lakes, respectively (Figures 6A–C). Furthermore, the average path length serves as a crucial indicator of the information exchange speed among the genera within the network, with smaller values indicating a faster response of the biological community to environmental changes. Consequently, the ecological network structure of bacterial communities in freshwater lakes demonstrated greater sensitivity than those in the brackish and salt lakes. The freshwater bacterial communities exhibited the highest modularity value (0.763), indicating their “small-world” nature and high interconnectivity (Jiao et al., 2023) (Figures 6D–F; Supplementary Table S4). The genera with high connectivity were observed within the same modules, highlighting the modular structure of the bacterial networks in freshwater lakes.

To further analyze the topological roles of each genus under different salinity gradients, we categorized the nodes in the network structure based on intra-module connectivity and inter-module connectivity. Module hubs and Connectors, located at the network’s center, are key nodes critical for maintaining structural stability. In the freshwater lakes, Bdellovibrionota (*Halobacteriovorax*), Cyanobacteria (*Schizothrix_LEGE_07164*), and Bacteroidota (*Cecembia*) were identified as the module hubs (Figure 6G). Notably, connectors were predominantly identified in the brackish and salt lakes. In the brackish lakes, they included Proteobacteria (*Thalassobaculum* and *Polycyclovorans*) and Bacteroidota (*Flavobacterium*), while in saline lakes, Actinobacteriota (*IMCC26207*), Thermotogota (*SC103*), and Desulfobacterota (*Desulfonatronovibrio*) were notable (Figures 6H,I).

## 4 Discussion

### 4.1 Environment-specific differences in changes in bacterial community structure governed by salinity

Lakes have host diverse, intricate microbial communities that are home to millions of bacteria, serving as pivotal sites for studying microbial community structures, which are crucial for maintaining the microecosystem functions (Yang et al., 2019). The results revealed that the freshwater lake bacterial communities exhibited the highest α-diversity, which was consistent with prior research (Liu et al., 2022) (Figure 2E). One potential explanation is that higher salinities screen the lake bacterial community. This coercive environment may influence the competitive abilities of bacterial communities for limited nutrients, prompting salt-tolerant species to thrive as fast-growing opportunists. Consequently, heightened competitive exclusion among these species may decrease bacterial community diversity (Ibelings et al., 2021; Jiao et al., 2023). Environmental conditions in freshwater lakes are conducive to bacterial survival, potentially leading to more rapid establishment of bacterial diversity (Mo et al., 2021). The reduced bacterial relative abundance in salt lakes supports the notion that salinity-driven environmental filtering selectively eliminates certain taxa (Jiao et al., 2023). Conversely, the brackish lakes exhibited a more uniform distribution of bacterial communities (with the highest mean value of Shannoneven’s index of about 0.61), likely influenced by varying salinity gradients that affected the bacterial dispersal (Figure 3A).

Additionally, the NMDS analysis confirmed the spatial variations in bacterial community structure across lake ecosystems with different salinity levels (Oren, 2011). Proteobacteria, Cyanobacteria, and Bacteroidota were predominantly present in the freshwater and salt lakes, accounting for approximately 45.71, 12.77, and 11.74% of the relative abundance, respectively (Figure 3B). Their dominance can be attributed to their high reproductive capacity, biodegradability, robust metabolism, and strong motility, rendering them less susceptible to predation than other bacterial taxa in lakes (Newton et al., 2011; Brinkmann et al., 2022). Typically, Firmicutes represents less than 1% of lakes, but its proportion is about 12.59% of freshwater lakes (Firmicutes proportions were 6.2 and 1.78% in brackish and salt lakes, respectively) suggesting a higher susceptibility to external influences than the brackish and salt lakes (Jiao et al., 2020) (Figure 3B). Moreover, the high-salinity environment further screened the bacterial community, which was particularly evident in the dominance of Halobacterota in the salt lakes. The relative abundance of Halobacterota reached 60.45%, which was significantly higher than that in the other lakes (only 0.42 and 0.66% in freshwater and salt lakes, respectively). Halobacterota possesses the specialized cellular structures that enables the adaptation to high-salt environments by regulating internal salinity concentrations (Bräuer et al., 2020). At the genus level, although the members of Proteobacteria and Cyanobacteria were widespread in freshwater and brackish lakes (e.g., *Acinetobacter*, *Limnobacter*, and *Candidatus_Aquiluna*), slight increases in salinity often coincided with relatively high nutrient levels (Supplementary Figure S1), potentially promoting the growth of genera such as *GKS98_freshwater_group* (Li et al., 2021; Liu et al., 2022) (Supplementary Table S3). Interestingly, the dominant bacterial genera *Halarchaeum*, *Halonotius* and *Halorubrum* in the salt lake were all classified as the members of Halobacterota, aligning with the phylum-level study findings. Similarly, the LDA analysis further confirmed that the notable differences in bacterial community composition across salinity gradients were attributed to the enrichment of biomarkers such as *Acinetobacter* (Figures 3D,E).

Nitrogen, a vital nutrient for microbial survival, converts TN into NH_4_^+^-N via electron transfer. Nitrogen was a key factor in explaining the bacterial community structure in the freshwater and brackish lakes, indicating heightened sensitivity to nitrogen levels in water bodies with lower relative salinity. In particular, the relative abundance of Proteobacteria, Firmicutes (*Enterococcus*), and Actinobacteriota (*Candidatus_Aquiluna*) was significantly promoted, probably due to their extensive metabolism and their ability to promote the transformation and degradation of substances, and the change in nitrogen content potentially accelerated bacterial community succession (Guan et al., 2022; Chen et al., 2023) (Figures 4C–E). SAL and COD, the predominant environmental factors in salt lakes, significantly influenced the growth of Halobacterota (*Halonotius*) (Figure 4E). This effect may result from the environment’s strong filtering effect on the bacterial communities, leading to the removal of sensitive taxa and retention of salt-tolerant bacteria. For example, Regulation of salt concentration within Halobacterota cells and structural adjustment of cell membranes for specialized adaptive mechanisms to tolerate high salt concentrations (Liu et al., 2016; Zhong et al., 2016; Jiao et al., 2022). Environmental heterogeneity selectively favors bacterial taxa adapted to strict conditions, thereby increasing their environmental resilience. The limited ecological niche in Salt Lake could increase species vulnerability to ecological selection, shaping the distinct bacterial taxa patterns, such as those observed in Halobacterota and its highly environment-responsive members (Logares et al., 2020; Luan et al., 2020) (Figure 5D).

### 4.2 Stochastic processes as key microbial community assembly processes

Ecosystem characteristics play a pivotal role in influencing species turnover and shifts in abundance (Karimi et al., 2018). Although significant expertise has been gained from previous studies analyzing bacterial community structures in lakes, exploring the mechanisms governing bacterial community assembly in aquatic ecosystems across varying salinity gradients remains an intriguing challenge.

We frequently utilized the null models to predict the bacterial community assembly processes, a practice supported by iCAMP findings (Langenheder and Lindstrom, 2019). In the Badanjilin Desert, most β-NTI scores for bacterial community assembly processes across various salinity gradients fell between-2 and + 2 on the observed dates, indicating community assembly is dominated by stochastic processes (Figures 5A,B). Furthermore, we demonstrated that although stochastic assembly was prevalent, its occurrence decreased in the salt lakes. This could be attributed to the higher population dispersal rate in freshwater ecosystems, which may strengthen the ecologically undominated effect in lake waters (Jiao et al., 2023). Moreover, the relatively low salinity in freshwater lakes is more conducive to the growth of a wide range of microorganisms, resulting in a more diverse bacterial community structure, promoting microbial growth and reproduction, and thereby enhancing the ecological undominated effect. In contrast, the strong control exerted on the bacterial communities in highly saline lakes enhanced the stability alongside effective screening, thereby significantly reducing the prevalence of ecologically undominated processes (Zeng et al., 2019). The dispersal limitation was the crucial ecological process that dominated the bacterial community in the lakes of the Badanjilin Desert. However, dispersal limitation significance underwent a shift from a dominant force in the freshwater lakes to a less dominant force in the brackish lakes (Figure 5C). This decline in dispersal limits can be attributed to several factors. Isolated ecosystems could exhibit greater dispersal constraints, whereas decreasing environmental complexity correlates with a reduced prevalence of dispersal limitations, thus amplifying the importance of homogeneous dispersal. In summary, the elevated salinity levels in salt lakes augmented the dispersal capabilities of bacterial communities (Wang et al., 2013; Huber et al., 2020). Regarding the deterministic processes, the heightened salinity levels correlated with an increased prevalence of such processes. Serving as a pivotal factor in altering bacterial community structures within salt lakes, increasing the salinity may intensify the deterministic processes on bacterial communities, while reducing the occurrence of stochastic processes. This has been evidenced by the favorable response of Halobacterota and its constituents (Hofmockel, 2014; Ning et al., 2020) (Figure 5).

The equilibrium of diverse constructive mechanisms within lake bacterial communities is mainly determined by environmental conditions, including habitat features and nutrient status. The variations in environmental conditions could regulate the bacterial community composition, indicating divergent phylogenetic adaptations among bacterial taxa under heterogeneous environmental conditions (Tripathi et al., 2018; Jiao et al., 2020). In this study, a robust correlation was observed between the β-NTI values of freshwater lake bacterial communities and several environmental factors, notably, phosphorus components (TP, DTP, and DIP), DO, and SAL. This finding was consistent with prior research that has highlighted the influence of elements such as phosphorus and SAL on bacterial community taxa (Supplementary Figure S2). The increase in nutrients within in the lakes of Badanjilin Desert (the COD content is about 107.28 mg/L, 248.28 mg/L and 1406.90 mg/L in freshwater, brackish and salt lakes, respectively) suggested a transition from stochastic to deterministic in the bacterial community construction process, which was characterized by heightened phylogenetic clustering tendencies (Rousk et al., 2010; Jiao et al., 2019). Furthermore, as the salinity increased, the impact of phosphorus on bacterial community construction decreased progressively (for example, the R2 value of the correlation between elemental phosphorus, represented by DTP, and β-NTI was about 0.96 in freshwater lakes, whereas it decreased to 0.04 and 0.008 in brackish and saltwater lakes, respectively), leading to the bacterial communities gradually converging towards a less stochastic state (Supplementary Figure S2).

### 4.3 Characterization of bacterial ecological network structure under different salinity features

A crucial yet neglected aspect of studying the bacterial community structures in the lakes with varying salinities is the examination of species interactions and the identification of keystone species. Our analysis of microbial networks across freshwater, brackish, and saline lakes revealed that the freshwater ecosystems exhibited the highest complexity in microbial networks, promoting stronger integration, possibly because of the unstable assembly state of the bacterial communities, which encouraged the heightened species interactions. This notion was further supported by the high cohesion among microorganisms, as indicated by their shortest average path lengths (Jordan, 2009; Lv et al., 2022) (Supplementary Table S4). The robust interactions between species within the lakes drove various ecological functions, characterized by the reciprocal or antagonistic interactions. The bacterial ecological network structure in the water of Badanjilin Desert Lake was predominantly characterized by positive correlations. These positive correlations became increasingly pronounced with increasing salinity. In the salt lake environment, positive correlations constituted over 95.00% of the bacterial ecological network structure. Salinity significantly affected the microbial interactions, resulting in heightened microbial activity and facilitating collaborative relationships among organisms. This contributed to the development of a highly intricate network of bacterial communities within the salt lakes (Brown et al., 2004).

As previously discussed, the environmental heterogeneity was intricately integrated into the modules of an ecological network. These modules represented closely related species with strong internal interactions, but limited the interactions with species from other modules, a pattern that was particularly pronounced in the freshwater lakes. Although our study did not focus on key taxa acting as connectors in freshwater lakes, species from dominant phyla families, such as *Cecembia* and *Schizothrix_LEGE_07164*, served as central hubs for information transfer within each module. These organisms significantly shaped the structure and function of the microbial communities (Figure 6G). The presence of *Polycyclovorans*, *Flavobacterium*, *IMCC26207*, *SC103*, and *Desulfonatronovibrio* as the connectors in brackish and saline lake waters was associated with increased functional associations (Figures 6H,I), enabling the adaptation to the narrow trophic spectrum of the lake and playing significant roles within their respective “small worlds.” The *Polycyclovorans* family, distinguished by bubble formation on cell surfaces and unipolar flagella, demonstrates a preference for aliphatic and aromatic hydrocarbon compounds as well as small organic acids (Gutierrez et al., 2013). The unique phenotypic and genotypic traits of the connectors presented their significance. The key taxa determined the community composition and metabolic functions, which were crucial for maintaining structural integrity. Their metabolic diversity influenced by environmental factors could enhance their dominance and play a pivotal role in lake microbial ecosystems (Banerjee et al., 2018).

## 5 Conclusion

In this study, we systematically investigated the distribution characteristics, symbiotic patterns, and assembly mechanisms of bacterial communities in the water bodies of Badanjilin Desert lakes, offering fresh insights into the bacterial community distribution under varying salinity gradients. Our findings indicated a reduction in bacterial community diversity with increasing salinity. Significant differences in bacterial community composition were observed among the different habitats, primarily attributed to the varying abundance of Halobacterota, constituting 60.45% of the salt lake community. Across the freshwater, brackish, and salt lakes, pH, COD, and Chla levels emerged as the dominant factors driving bacterial community composition in the water column. Fluctuating salinity levels in lakes induced the shifts in the mechanisms governing bacterial community assembly. Despite the stochastic dominance in the bacterial community assembly in the lakes of the Badanjilin Desert, the influence of high-salinity screens against undominated occurrences emphasized the growing importance of homogeneous dispersal. Environmental preferences may dictate varying assembly patterns, with phosphorus and Chl a levels regulating the transition between deterministic and stochastic processes in bacterial community formation. Furthermore, the differences in key species within bacterial community coexistence networks were observed across water bodies with different salinities. The freshwater lake networks could exhibit higher complexity, promoting the development of more sophisticated modularized structures. In conclusion, this study offered a fresh perspective on how environmental variations across lakes with diverse salinity gradients influenced the characterization and construction of bacterial communities. These insights shed light on the response of lake microorganisms to ecological processes.

## Data Availability

The original contributions presented in the study are included in the article/Supplementary material, further inquiries can be directed to the corresponding author.
