# Supplementary material for: Characterization of bacterial community dynamics dominated by salinity in lakes of the Inner Mongolian Plateau, China
Source: Front Microbiol. 2024 Aug 21;15:1448919. doi: 10.3389/fmicb.2024.1448919 (PMC11371557; doi:10.3389/fmicb.2024.1448919)

**Supplementary Table1 Diversity indices of bacterioplankton in Freshwater Lakes, Brackish Lakes, and Salt Lakes**

| Type | Samples | Chao | Shannoneven | Shannon |
| --- | --- | --- | --- | --- |
| Freshwater Lakes | A7 | 1349.142259 | 0.465963 | 3.296275 |
|  | A8 | 1334.695122 | 0.594142 | 4.244791 |
|  | A14 | 826.5107910 | 0.733893 | 4.883437 |
|  | Mean | 1170.000000 | 0.598 | 4.142000 |
| Brackish Lakes | A1 | 1305.224806 | 0.640192 | 4.503926 |
|  | A2 | 956.4603170 | 0.561213 | 3.771475 |
|  | A6 | 659.7567570 | 0.367507 | 2.375782 |
|  | A9 | 540.8000000 | 0.634504 | 3.960714 |
|  | A10 | 878.1573030 | 0.651143 | 4.393647 |
|  | A13 | 763.6375000 | 0.777567 | 5.132895 |
|  | Mean | 850.7000000 | 0.6054 | 4.023000 |
| Salt Lakes | A3 | 577.6363640 | 0.615286 | 3.832315 |
|  | A4 | 381.1184210 | 0.404061 | 2.383918 |
|  | A5 | 793.8163270 | 0.438558 | 2.862246 |
|  | A11 | 378.4516130 | 0.615856 | 3.653410 |
|  | A12 | 325.7000000 | 0.572602 | 3.302950 |
|  | Mean | 491.3000000 | 0.5293 | 3.207000 |

**Supplementary Table 2 Percentage of bacterial phyla in Freshwater Lakes, Brackish Lakes, and Salt Lakes**

| Phylum | Freshwater Lakes(%) | | | | Brackish Lakes(%) | | | | | | | Salt Lakes(%) | | | | | |
| --- | --- | --- | --- | --- | --- | --- | --- | --- | --- | --- | --- | --- | --- | --- | --- | --- | --- |
|  | A7 | A8 | A14 | Mean | A1 | A2 | A6 | A9 | A10 | A13 | Mean | A3 | A4 | A5 | A11 | A12 | Mean |
| Proteobacteria | 58.09 | 50.32 | 26.76 | 45.06 | 27.11 | 49.61 | 73.51 | 36.16 | 67.53 | 24.20 | 46.35 | 59.17 | 22.48 | 22.61 | 0.19 | 0.03 | 20.90 |
| Halobacterota | 1.07 | 0.12 | 0.07 | 0.42 | 0.07 | 0.09 | 0.03 | 0.36 | 0.01 | 3.38 | 0.66 | 0.26 | 61.60 | 52.29 | 95.11 | 92.99 | 60.45 |
| Bacteroidota | 2.59 | 2.18 | 21.77 | 8.85 | 10.10 | 23.02 | 1.44 | 33.95 | 4.37 | 14.82 | 14.62 | 21.90 | 6.09 | 12.47 | 1.12 | 2.79 | 8.87 |
| Cyanobacteria | 31.31 | 5.38 | 14.67 | 17.12 | 26.16 | 3.92 | 1.76 | 2.42 | 0.53 | 15.64 | 8.41 | 1.39 | 0.17 | 0.52 | 0.00 | 0.00 | 0.42 |
| Firmicutes | 3.15 | 31.03 | 3.58 | 12.59 | 5.26 | 9.09 | 5.27 | 0.41 | 3.48 | 13.70 | 6.20 | 2.53 | 3.26 | 3.09 | 0.01 | 0.01 | 1.78 |
| Actinobacteriota | 0.75 | 1.30 | 3.07 | 1.71 | 11.01 | 11.67 | 13.13 | 9.03 | 0.75 | 3.38 | 8.16 | 12.77 | 4.05 | 2.52 | 0.00 | 0.00 | 3.87 |
| Verrucomicrobiota | 0.47 | 3.56 | 14.22 | 6.08 | 5.33 | 0.32 | 0.52 | 16.08 | 0.16 | 7.88 | 5.05 | 1.05 | 0.32 | 1.33 | 0.03 | 0.00 | 0.55 |
| Planctomycetota | 0.21 | 0.71 | 8.81 | 3.24 | 9.52 | 0.20 | 0.06 | 0.31 | 0.19 | 6.20 | 2.75 | 0.06 | 0.03 | 0.20 | 0.00 | 0.01 | 0.06 |
| Campilobacterota | 0.19 | 0.32 | 0.01 | 0.17 | 0.02 | 0.05 | 0.03 | 0.01 | 20.54 | 0.00 | 3.44 | 0.01 | 0.41 | 0.49 | 0.00 | 0.00 | 0.18 |
| Chloroflexi | 0.12 | 0.61 | 2.55 | 1.09 | 0.54 | 0.17 | 0.20 | 0.01 | 0.19 | 8.45 | 1.59 | 0.02 | 0.00 | 0.01 | 0.00 | 0.00 | 0.01 |
| Patescibacteria | 0.56 | 1.77 | 0.67 | 1.00 | 0.24 | 0.55 | 2.68 | 0.12 | 0.09 | 0.87 | 0.76 | 0.08 | 0.08 | 0.22 | 0.01 | 0.04 | 0.09 |
| Nanohaloarchaeota | 0.06 | 0.00 | 0.01 | 0.02 | 0.00 | 0.00 | 0.00 | 0.01 | 0.00 | 0.17 | 0.03 | 0.00 | 0.04 | 0.00 | 2.56 | 3.75 | 1.27 |
| Desulfobacterota | 0.18 | 0.55 | 0.15 | 0.29 | 0.69 | 0.38 | 0.23 | 0.34 | 0.59 | 0.15 | 0.40 | 0.07 | 0.27 | 1.02 | 0.10 | 0.07 | 0.31 |
| Spirochaetota | 0.09 | 0.03 | 0.06 | 0.06 | 1.14 | 0.03 | 0.05 | 0.00 | 0.03 | 0.01 | 0.21 | 0.02 | 0.05 | 0.13 | 0.00 | 0.00 | 0.04 |
| Myxococcota | 0.01 | 0.03 | 1.08 | 0.37 | 0.07 | 0.01 | 0.06 | 0.01 | 0.05 | 0.01 | 0.04 | 0.00 | 0.00 | 0.02 | 0.00 | 0.00 | 0.01 |
| others | 1.13 | 2.09 | 2.52 | 1.91 | 2.75 | 0.92 | 1.02 | 0.78 | 1.48 | 1.14 | 1.35 | 0.66 | 1.14 | 3.08 | 0.86 | 0.31 | 1.21 |

**Supplementary Table 3 Percentage of bacterial genera in Freshwater Lakes, Brackish Lakes, and Salt Lakes**

| Genus | Freshwater Lakes | | | | Brackish Lakes | | | | | | | Salt Lakes | | | | | |
| --- | --- | --- | --- | --- | --- | --- | --- | --- | --- | --- | --- | --- | --- | --- | --- | --- | --- |
|  | A7 | A8 | A14 | Mean | A1 | A2 | A6 | A9 | A10 | A13 | Mean | A3 | A4 | A5 | A11 | A12 | Mean |
| *Halarchaeum* | 0.08596 | 0.00860 | 0 | 0.03152 | 0.02579 | 0.02865 | 0 | 0.04011 | 0 | 0 | 0.01576 | 0.18911 | 59.68597 | 52.19621 | 0 | 0 | 22.41426 |
| *Halonotius* | 0.07736 | 0 | 0.01719 | 0.03152 | 0.00287 | 0.00573 | 0.00860 | 0.02865 | 0 | 0.67906 | 0.12082 | 0.00287 | 0.81946 | 0.00860 | 45.69497 | 52.88101 | 19.88139 |
| *unclassified_f__Rhodobacteraceae* | 1.13464 | 2.16326 | 6.50984 | 3.26925 | 10.79052 | 7.52127 | 1.16616 | 7.70179 | 2.53574 | 1.81370 | 5.25486 | 27.21125 | 5.24054 | 2.55294 | 0.00573 | 0 | 7.00209 |
| *GKS98_freshwater_group* | 0.10601 | 0.12894 | 0.83379 | 0.35625 | 0.07450 | 11.05986 | 57.15596 | 1.89106 | 0 | 0 | 11.69690 | 1.17762 | 0 | 0.00287 | 0 | 0 | 0.23610 |
| *unclassified_f__Comamonadaceae* | 1.15183 | 13.37784 | 0.55872 | 5.02946 | 0.29512 | 21.63835 | 4.51276 | 0.28652 | 3.83657 | 1.89106 | 5.41006 | 1.22346 | 0.00573 | 0 | 0 | 0 | 0.24584 |
| *Acinetobacter* | 0.95986 | 27.05080 | 3.75920 | 10.58995 | 0.02006 | 0.04871 | 5.53279 | 0.89396 | 0.29512 | 7.37515 | 2.36097 | 0.01146 | 0 | 0 | 0.00573 | 0 | 0.00344 |
| *Halorubrum* | 0.38394 | 0.00573 | 0.04011 | 0.14326 | 0 | 0.00573 | 0.01433 | 0.21203 | 0 | 1.95983 | 0.36532 | 0.02006 | 0.51574 | 0.01433 | 23.15120 | 16.96800 | 8.13387 |
| *Candidatus_Aquiluna* | 0.07450 | 0.54153 | 0.64182 | 0.41928 | 0 | 7.03418 | 12.58990 | 3.74774 | 0 | 0.24928 | 3.93685 | 6.89379 | 2.13747 | 1.39538 | 0 | 0 | 2.08533 |
| *Limnobacter* | 34.58067 | 0.10028 | 0.08309 | 11.58801 | 0 | 0 | 0.04584 | 0.12607 | 0.03438 | 0 | 0.03438 | 0 | 0 | 0 | 0 | 0 | 0 |
| *Flavobacterium* | 0.40973 | 0.81087 | 0.05444 | 0.42501 | 0.09169 | 11.06845 | 0.06877 | 17.91926 | 0.01146 | 2.38389 | 5.25725 | 1.01143 | 0.00573 | 0 | 0 | 0 | 0.20343 |
| *Halomonas* | 0.35243 | 0.02579 | 0 | 0.12607 | 0.63322 | 0.12321 | 0.14040 | 0.32664 | 0.06877 | 0.20343 | 0.24928 | 18.11409 | 5.16318 | 6.61299 | 0.03725 | 0 | 5.98550 |
| *Tychonema_CCAP_1459-11B* | 25.74138 | 0.00860 | 0.00287 | 8.58428 | 0 | 0 | 0.06017 | 0 | 0 | 0 | 0.01003 | 0 | 0 | 0 | 0 | 0 | 0 |
| *norank_f__norank_o__Chloroplast* | 3.19475 | 3.00564 | 3.96550 | 3.38863 | 2.00854 | 2.99991 | 0.96559 | 2.29220 | 0.36675 | 1.22346 | 1.64274 | 1.12318 | 0.08596 | 0.30085 | 0 | 0 | 0.30200 |
| *Cyanobium_PCC-6307* | 1.16043 | 0.19770 | 3.24919 | 1.53577 | 15.89066 | 0.10601 | 0.04011 | 0.02865 | 0 | 0.55013 | 2.76926 | 0.06304 | 0 | 0 | 0 | 0 | 0.01261 |
| *Alishewanella* | 1.01716 | 0.02006 | 0 | 0.34574 | 0 | 0.02292 | 0.04011 | 0.01146 | 19.26019 | 0.02292 | 3.22627 | 0 | 0 | 0 | 0 | 0 | 0 |
| *Luteolibacter* | 0.16905 | 0.68193 | 0.83952 | 0.56350 | 0.93121 | 0.09455 | 0.02865 | 15.78178 | 0.00573 | 0.31231 | 2.85904 | 0.48423 | 0 | 0 | 0 | 0 | 0.09685 |
| *unclassified_f__Microbacteriaceae* | 0.08023 | 0.12894 | 0.44984 | 0.21967 | 8.45248 | 2.05438 | 0.27220 | 2.40108 | 0.03725 | 0.08309 | 2.21675 | 3.21481 | 0.04584 | 0 | 0 | 0 | 0.65213 |
| *Sulfurimonas* | 0.01719 | 0 | 0 | 0.00573 | 0 | 0.00573 | 0.00860 | 0 | 16.90496 | 0 | 2.81988 | 0 | 0.03725 | 0.07736 | 0 | 0 | 0.02292 |
| *Psychroflexus* | 0.01433 | 0.00573 | 0 | 0.00669 | 0.13467 | 0.01433 | 0.01146 | 0 | 0 | 0.01719 | 0.02961 | 9.14014 | 2.36956 | 4.91676 | 0 | 0 | 3.28529 |
| *Candidatus_Aquirestis* | 0 | 0 | 10.07134 | 3.35711 | 0 | 0 | 0 | 0 | 0 | 4.37523 | 0.72921 | 0 | 0 | 0 | 0 | 0 | 0 |
| *LD29* | 0.01433 | 0.00860 | 7.65881 | 2.56058 | 0.80227 | 0 | 0 | 0 | 0 | 4.05719 | 0.80991 | 0.00573 | 0 | 0 | 0 | 0 | 0.00115 |
| *Spiribacter* | 0.10888 | 0 | 0 | 0.03629 | 0.00573 | 0.02292 | 0.00573 | 0.02865 | 0 | 0.02579 | 0.01480 | 4.20332 | 4.03427 | 2.93115 | 0 | 0 | 2.23375 |
| *Algoriphagus* | 0.06877 | 0.04298 | 0.11461 | 0.07545 | 0.04011 | 5.25200 | 0.45557 | 4.30647 | 0.27793 | 0.00287 | 1.72249 | 0.41833 | 0 | 0.00573 | 0 | 0 | 0.08481 |
| *norank_f__Pirellulaceae* | 0.09169 | 0.18051 | 1.24638 | 0.50619 | 7.61296 | 0.00573 | 0.02006 | 0.00860 | 0.01146 | 1.14896 | 1.46796 | 0 | 0 | 0.02579 | 0 | 0 | 0.00516 |
| *Trichococcus* | 0 | 9.21750 | 0.20916 | 3.14222 | 0 | 0.12607 | 0 | 0 | 0.59884 | 0 | 0.12082 | 0 | 0 | 0 | 0 | 0 | 0 |
| *norank_f__MWH-UniP1_aquatic_group* | 0.03438 | 0 | 4.46406 | 1.49948 | 2.73058 | 0 | 0.78508 | 1.50712 | 0 | 0.20916 | 0.87199 | 0.00287 | 0 | 0 | 0 | 0 | 0.00057 |
| *Fusibacter* | 0.00287 | 0.02006 | 0 | 0.00764 | 0.00860 | 0.01433 | 0.01146 | 0 | 0.00573 | 9.22896 | 1.54485 | 0.01433 | 0.11748 | 0.23495 | 0 | 0 | 0.07335 |
| *Planktothrix_NIVA-CYA_15* | 0.02865 | 0 | 0 | 0.00955 | 0.01146 | 0.00573 | 0.00287 | 0 | 0.06590 | 9.48683 | 1.59547 | 0 | 0 | 0.00573 | 0 | 0 | 0.00115 |
| *Haloquadratum* | 0.02006 | 0 | 0.00287 | 0.00764 | 0 | 0.00287 | 0 | 0 | 0 | 0 | 0.00048 | 0 | 0.05444 | 0 | 4.03427 | 4.93682 | 1.80511 |
| *Halobellus* | 0.01719 | 0 | 0 | 0.00573 | 0 | 0 | 0 | 0.00287 | 0 | 0.04011 | 0.00716 | 0 | 0.08882 | 0 | 4.19186 | 4.59299 | 1.77473 |
| *Others* | 14.50388 | 22.42629 | 19.06249 | 18.66422 | 18.43500 | 7.75336 | 8.41810 | 12.21741 | 23.17126 | 23.51222 | 15.58456 | 8.34933 | 6.96542 | 12.51254 | 6.96542 | 5.48981 | 8.05650 |

**Supplementary Table 4 Ecological network topology characteristics of bacterial communities in Freshwater Lakes, Brackish Lakes, and Salt Lakes**

| Indicators | Freshwater Lakes | Brackish Lakes | Salt Lakes |
| --- | --- | --- | --- |
| Average degree | 91.699 | 31.932 | 65.355 |
| Network diameter | 1 | 5 | 10 |
| Density | 0.184 | 0.064 | 0.150 |
| Modularization | 0.763 | 0.713 | 0.713 |
| Average path length | 1 | 2.734 | 2.154 |
| Positive correlation | 64.55% | 75.75% | 95.50% |
| Negative correlation | 35.45% | 24.25% | 4.50% |
| Edges | 22880 | 7984 | 14281 |

**Supplementary Fig. 1. Environmental characteristics in Freshwater Lakes, Brackish Lakes, and Salt Lakes**


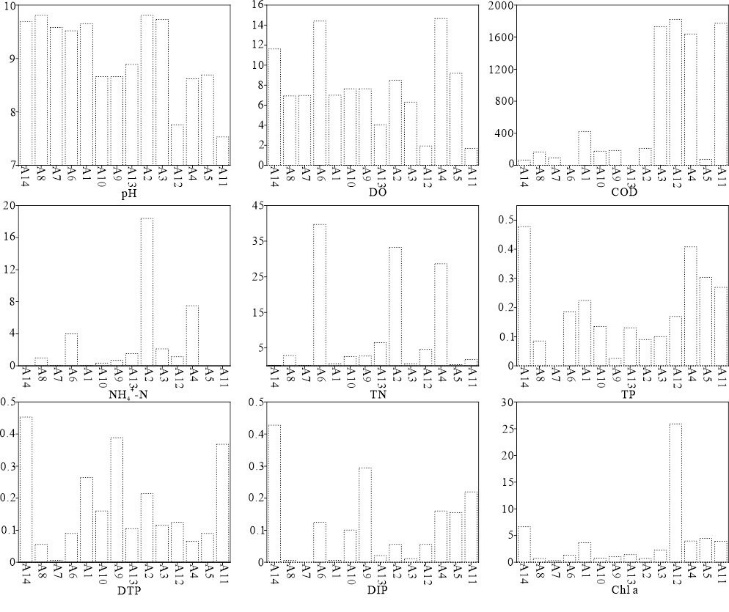


**Supplementary Fig. 2. Relationships between β-NTI of bacterial communities and environmental parameters in the water bodies of Freshwater lakes, Brackish Lakes, and Salt Lakes**


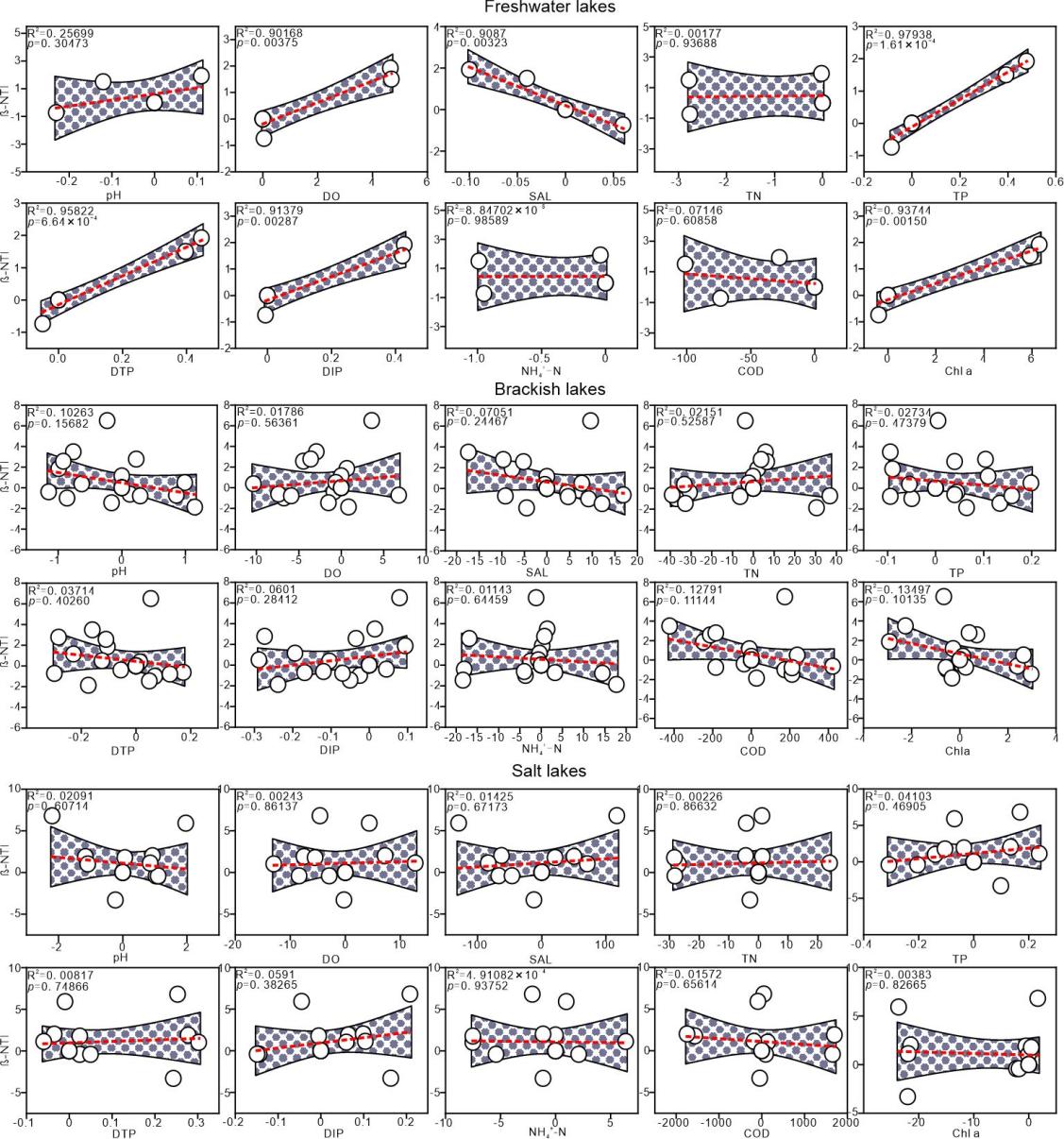

Supplement: Supplementary file 1 [file Data_Sheet_1.docx]
